# Building and Repairing Trust in Chatbots: The Interplay Between Social Role and Performance During Interactions

**DOI:** 10.3390/bs16010118

**Published:** 2026-01-14

**Authors:** Yi Mou, Xiaoyu Ye, Wenbin Ma

**Affiliations:** School of Media and Communication, Shanghai Jiao Tong University, 800 Dongchuan Road, Minhang District, Shanghai 200240, China; yimou@sjtu.edu.cn (Y.M.); 2489erin@sjtu.edu.cn (X.Y.)

**Keywords:** chatbot, AI, trust, investment/trust game, trust repair, social role

## Abstract

Trust (or distrust) in artificial intelligence (AI) is a critical research topic, given AI’s pervasive integration across societal domains. Despite its significance, scholarly attention to process-based learned trust in AI remains limited. To address this gap, this study designed a virtual non-fungible token (NFT) investment task, featuring seven rounds of risk decision-making scenarios, to simulate an investment/trust game to explore participants’ multifaceted trust under the influence of different chatbots’ social role. The findings suggested the chatbot’s social role had a significant impact on participants’ trust behaviors and perceptions over time. Trust in the two chatbot types diverged until the system-induced failures occurred. The friend-like chatbot elicited a higher level of behavioral trust than the servant-like counterpart. During those trust-damaging moments, the friend-like chatbot proved more effective in mitigating trust erosion and facilitating trust repair, as evidenced by relatively stable investment behaviors. The findings reinforce the notion that friendship with AI can function as a relational buffer, softening the impact of trust violations and facilitating smoother trust recovery.

## 1. Introduction

To trust or not to trust technology? This has been a long-standing question, and has evolved into various forms. In contemporary discourse, the concept of “trustworthy artificial intelligence (AI)” has emerged as a critical framework, underscoring the imperative of trustworthiness in AI systems (Kaur et al., 2022), given their ubiquitous integration across societal sectors.

Existing scholarship on trust in AI chatbots has predominantly centered on the attributes of chatbots and user characteristics (see a review in Rheu et al., 2021). Research indicates that chatbot design elements such as vocal features and communication styles significantly influence user trust; user demographics, personality traits, and usage contexts moderate these effects (e.g., Torre et al., 2019; Wu et al., 2024). Notwithstanding, trust is fundamentally a dynamic process, and chatbot performance is expected to exert a substantial influence on evolving user trust. Generally speaking, consistent trustee performance fosters trust, whereas performance discrepancies erode trust (Yang & Holzer, 2006). Take self-driving cars as an example: advancements in autonomous vehicle technology often engender public trust, yet high-profile crash incidents can greatly undermine confidence in the safety of these systems (Hawkins, 2024).

Given the paucity of research on process-based or learned trust in AI chatbots, this study designed an investment/trust game simulation to explore participants’ multifaceted trust constructs. Additionally, recognizing the impact of chatbot anthropomorphic features on user perception and experience, this study attempts to examine the interplay between social role attribution and performance during interactions in shaping learned trust by employing a laboratory experiment design.

## 2. Literature Review

### 2.1. Learned Trust in Chatbots

With the rapid development of AI and the proliferation of intelligent conversational agents, chatbots have become a prevalent interface for human–machine interaction. In domains ranging from customer service to mental health support and education, it is expected to build and maintain trust in chatbots in dynamic, task-specific contexts (Omarov et al., 2023). This growing reliance on conversational AI calls for a nuanced understanding of trust as a fluid, context-sensitive process, rather than a static disposition.

Trust, commonly recognized as a foundational element of human interaction, refers to a psychological state where individuals accept vulnerability based on positive expectations of others’ intentions or behaviors (Rousseau et al., 1998). Trust comprises cognitive evaluations (i.e., trust beliefs in competence, benevolence, and integrity) and behavioral intentions (i.e., willingness to take risks) (Mayer et al., 1995; Xie, 2024). McAllister (1995) differentiates cognitive trust and affective trust, as the former relies on rational assessments of reliability, and the latter is emotional bonds with each other during interpersonal cooperation in organizations. In addition, behavioral trust is the result of both cognitive and affective trust, reflecting in actual behaviors that demonstrate trust (D. Johnson & Grayson, 2005).

Three types of trust in artificial agents have been identified by Grodzinsky et al. (2020): dispositional trust (a general tendency to trust), situational trust (based on context), and learned trust (shaped by direct interaction). In particular, learned trust refers to the trust that emerges and evolves through interaction with an AI system, and it is further subdivided into initial learned trust and dynamic learned trust (Collins & Juvina, 2021). Initial learned trust is typically shaped by pre-existing expectations, such as brand perception, interface design, and prior experiences. In contrast, dynamic learned trust evolves continuously as users interpret real-time system behaviors, assess reliability, and make interactional adjustments (J. D. Lee & See, 2004).

Although researchers have recognized the importance of dynamic trust, empirical investigations often lack the temporal sensitivity required to capture its development over time. Most studies employ cross-sectional methods, using single-point questionnaires or post-task surveys, which fail to reflect the fluctuations of trust throughout the interaction (Diederich et al., 2020). For example, trust may peak when a chatbot provides a helpful response and decline sharply when it makes an error—nuances are lost in static measurement paradigms. Even in experimental settings, trust is frequently measured only at the end of a session (Przegalińska et al., 2019), neglecting the dynamic, iterative nature of human-chatbot relationships. This lack of temporal granularity in trust research is especially problematic in high-stakes or ambiguous scenarios where trust decisions are closely tied to user vulnerability and perceived risk. Mayer et al. (1995) posited that trust becomes most salient and behaviorally meaningful when users must take risks—such as relying on a chatbot for sensitive information or critical decisions. In this light, M. Johnson and Bradshaw (2021) introduced a behavioral framework that ties trust directly to risk exposure, suggesting that users express trust primarily through their willingness to act on AI suggestions in uncertain environments. This framework supports the notion that trust should be modeled dynamically—across time intervals and behavioral episodes.

Empirical studies on learned trust in chatbots are still in scarcity. A majority of the trust assessments still rely on subjective self-reports, which are prone to retrospective bias and fail to account for role-specific trajectories of trust development and repair. There is a pressing need for experimental designs that incorporate real-time data collection, such as trust ratings at each conversational turn or behavioral shifts in response to role-incongruent behavior. By combining trust metrics with observable risk-taking behavior across chatbot roles, research can offer richer evidence of how dynamic learned trust manifests in practice.

### 2.2. Damaging and Repairing Trust in Chatbots

Trust development is a gradual process shaped by cognitive, affective, and structural factors. Cognitive mechanism involves positive attributions, such as transparency in decision-making which would enhance perceptions of integrity (Hardin, 2002). Affective-social mechanism relies on norms like reciprocity and symbolic acts (e.g., apologies) to maintain relational balance (Uslaner, 2002). Structural safeguards, such as contracts or third-party certifications, reduce perceived risks, with digital contexts emphasizing algorithmic transparency and user interfaces (Robbins, 2016). While those foundations are disrupted, trust is eroded, triggering cognitive doubts (e.g., questioning competence and/or integrity), emotional distress (e.g., anger and disillusionment), and behavioral withdrawal (e.g., reduced cooperation, retaliation) (Lewicki et al., 1998; Xie, 2024). The damage of trust has been categorized into three types: Competence-based such as inability-related failures, integrity-based such as intentional dishonesty, and benevolence-based such as neglect-induced harm (P. H. Kim et al., 2004; Yao et al., 2012). They highlight its fragility and the complexity of repair needs. Spillover effect further amplifies damage, as a competence failure in one domain may taint unrelated integrity perceptions (Uslaner, 2002). This dynamic vulnerability underscores the urgency of understanding how trust, once broken, can be systematically repaired, as emphasized by Robbins (2016).

In turn, restoring trust requires addressing cognitive, emotional, and structural dimensions. At interpersonal level, multiple theoretical frameworks have been developed, including attribution reshaping causal inferences by blaming external and unstable factors (Weiner, 1985), social equilibrium which rebalances norms through apologies or penance (Goffman, 1967), and structural mechanisms instituting audits/penalties to deter future violations (Gillespie & Dietz, 2009), etc. However, trust repair strategies vary in contexts. For instance, apologies usually work for competence violations but may backfire for integrity breaches, while denials risk insincerity (P. H. Kim et al., 2004). Substantive actions like compensation or self-punishment (e.g., resignations) signal commitment to change (Hardin, 2002).

### 2.3. The Effect of Chatbots’ Social Role

Anthropomorphism, the tendency to attribute human-like characteristics to non-human agents (Epley et al., 2007), profoundly influences human–machine interactions, particularly in shaping how users perceive and trust chatbots. Three dimensions of anthropomorphic features in human–machine communication have been identified: human identity, verbal cues, and nonverbal cues (Seeger et al., 2021). Grounded in the Computers Are Social Actors (CASA) paradigm, which posits that humans apply social norms to machines (Nass & Moon, 2000), the social role assigned to a chatbot—whether as a servant, friend, partner, or assistant—shapes user interactions by invoking behavioral expectations akin to human relationships. Recent research suggests that the nature and pace of learned trust development may vary significantly depending on the chatbot’s social role. For example, friend-like chatbots, which emphasize socio-emotional connection through informal language, personalized responses, and empathic cues, have been found to accelerate the accumulation of affective trust, especially during early interactions (Huh et al., 2023). In contrast, servant-like chatbots, which use formal language and adopt task-oriented roles, are more likely to build behavioral trust over time by consistently demonstrating competence and goal efficiency (Chattaraman et al., 2019; Youn & Jin, 2021). These differences suggest that dynamic trust is not only interaction-dependent but also role-contingent, aligning with the CASA paradigm.

Two social roles of chatbots are commonly selected: friend (or partner) and servant (or assistant), as they reflect two ends of the relationship spectrum from equality to subordinance (Youn & Jin, 2021; Zhang, 2023). Friend-like chatbots, which simulate companionship through informal language, emotional tone, and empathetic responses, tend to elicit higher levels of affective trust that is defined as the emotional willingness to be vulnerable (McAllister, 1995). In their study with 158 college students interacting with AI voice assistants, A. Kim et al. (2019) discovered that friend-like AI evoked greater perceptions of warmth and pleasure than servant-like AI. Gupta and Nagar (2024) found that participants interacting with friend-like anthropomorphic voice assistants reported significantly higher affective trust, measured through self-reported closeness and willingness to engage in non-task-related conversations. These chatbots, characterized by informal language and empathetic responses, created stronger emotional bonds, aligning with McAllister’s (1995) definition of affective trust as emotional vulnerability. For behavioral trust, friend-like chatbots also demonstrated higher compliance in low-risk tasks, such as sharing personal preferences, due to perceived social rapport. In contrast, servant-like chatbots, while effective in task execution, lacked the relational cues necessary to elicit immediate behavioral trust, as users viewed them primarily as tools rather than companions (Schweitzer et al., 2019).

In terms of trust building, recent findings suggest that friend-like chatbots may foster trust more quickly than servant-like ones by leveraging social presence, emotional attunement, and relational cues such as humor, empathy, and informal language (Huh et al., 2023). This indicates that friend-like chatbots may indeed build trust more rapidly than servant-like counterparts, especially when affective trust is prioritized by users. However, coming with a cost, when trust violations occur, the damage may also be more pronounced. Friend-like chatbots tend to evoke stronger emotional involvement, which intensifies users’ reactions to perceived breaches of expectations. When a friend-like chatbot fails to meet relational or emotional norms such as being unresponsive, dismissive, or inauthentic, users may interpret the violation as a personal betrayal, resulting in higher levels of both affective trust damage (e.g., feelings of disappointment or hurt) and behavioral trust damage (e.g., disengagement, reduced cooperation). Research on relational trust violations indicates that breaches involving socio-emotional expectations are often perceived as more painful and lead to more significant withdrawal behaviors than instrumental or competence-based failures (P. H. Kim et al., 2004).

When it comes to trust repair, the friend-like chatbot’s social orientation may offer a unique advantage. Affective strategies such as personalized apologies and reaffirmation of the relationship can enhance users’ perceptions of warmth and relational closeness. Research shows that social-oriented communication by chatbots increases user satisfaction, especially for those with high attachment anxiety, by fostering emotional connection (Xu et al., 2022), suggesting such affective strategies may also contribute to trust restoration in emotionally sensitive interactions. Users may be more forgiving toward friend-like chatbots when violations are framed as misunderstandings rather than failures of competence, allowing for faster affective trust repair.

Based on the literature review, five hypotheses have been postulated:

**H1.**  *The social role of a chatbot influences users’ initial trust toward the chatbot. Specifically, compared to a servant-like chatbot, a friend-like chatbot will elicit higher levels of (a) behavioral trust, and (b) affective trust.*

**H2.**  *The social role of a chatbot influences users’ trust building toward the chatbot. Specifically, compared to a servant-like chatbot, a friend-like chatbot will elicit faster (a) behavioral and (b) affective trust building.*

**H3.**  *The social role of a chatbot influences users’ trust damaging toward the chatbot. Specifically, compared to a servant-like chatbot, a friend-like chatbot will elicit higher levels of (a) behavioral and (b) affective trust damaging.*

**H4.**  *The social role of a chatbot influences users’ trust repairing toward the chatbot. Specifically, compared to a servant-like chatbot, a friend-like chatbot will gain faster (a) behavioral and (b) affective trust repair after the trust damage.*

**H5.**  *The social role of a chatbot influences users’ overall cognitive trust toward the chatbot. Specifically, compared to a servant-like chatbot, a friend-like chatbot will obtain higher levels of cognitive trust.*

## 3. Method

### 3.1. Procedure

To simulate a dynamic trust building, damaging, and repairing process, this study adapted the investment/trust game widely used in economy and social psychology fields (e.g., Lunawat, 2013). In this paradigm, a trustor (participant) decides how much of an endowed resource to entrust to a trustee (chatbot) for investment, with final payoffs contingent on the outcome, effectively measuring trust as willingness to take risks (Houser et al., 2010; Kräkel, 2008). The experiment employed the context of a virtual non-fungible token (NFT) investment task, featuring seven rounds of risk decision-making scenarios. The NFT context was chosen due to its relative novelty and simplicity, so participants will not be influenced unduly by any existing experience with traditional forms of investment such as stock trading. In each round, participants were allocated 100 virtual coins and needed to determine the amount to delegate based on the investment analysis provided by a chatbot named Tobi. After each round of investment was completed, participants were asked to rate their trust level toward Tobi. The experiment simulated trust damage and repair processes by pre-set investment losses in the 4th and 6th rounds, allowing for dynamic tracking of trust fluctuations.

After securing approval from the institutional review board of Shanghai Jiao Tong University (no. H20240527I), a lab experiment was conducted in this university in Eastern China in October 2024. Upon the arrival of each participant, s/he was greeted at the entrance of the main lab area by a research assistant, and then led to a lab room, equipped with a table, a chair, and a laptop. After obtaining the written consent form, each participant first watched a tutorial video, introducing a fictitious AI company named *High Fidelity* as a globally leading art investment company featuring a cutting-edge AI algorithm to maximize gains from investing in NFT art. The story was framed as follows: *High Fidelity* started to enter the Chinese market and recruited the Center for Future Media and Human-Machine Communication at this university to test the functionality of the metaverse *Omnisphere* and its affiliated algorithm. The chatbot Tobi worked as a guide to the system and provided investment suggestions for users. Each participant could choose whether, and to what extent, to follow Tobi’s suggestions. Their investment amounts reflected their behavioral trust toward Tobi, as indicated in previous studies (e.g., Buchan et al., 2008). To encourage participants to take the investment seriously, an additional incentive was emphasized in addition to the gain from the investment itself: The top 5 players would have the chance of becoming the campus ambassador of *High Fidelity* and being awarded an official bonus.

The two-minute-and-44-second-long tutorial video consists of both the introduction of the NFT art investment (the cover story) and guidelines to complete the experimental procedure. Once each participant finished watching the video and had no further questions, the assistant left the room to leave the participant to complete the experiment. During the 7-round investment, questions were asked as part of the measurement (see Section 3.4). After the investment was over, each participant was asked to complete an online questionnaire. Once the whole experimental procedure end, the assistant came in, debriefed and thanked the participant. Each participant was rewarded either extra credit or RMB 50 Yuan in cash according to their preference.

**Figure 1:**
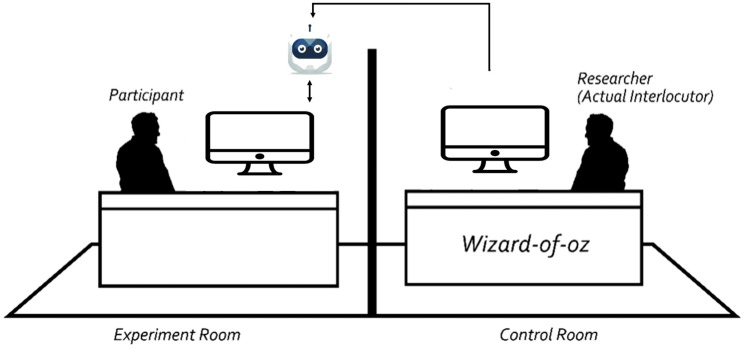
The Wizard-of-Oz Experimental Design.

The study adopted the *Wizard of Oz* method, in which trained operators simulated AI conversations in real-time on *Feishu* platform, a popular Chinese collaboration and management platform with messaging, video conferencing, and cloud documents in a professional business setting. In a nearby lab room, a research assistant chatted with the participant as Tobi in the whole experimental procedure. Experimental manipulations were implemented through standardized dialogue scripts (see Section 3.3). Given that chatbot avatars influence users’ trust level (Bae et al., 2023), this study designed a generic chatbot avatar. To avoid the influence of gender-specific chatbot designs, the avatar for the chatbot Tobi was designed to appear more robotic than human-like, as shown in Figure 1.

### 3.2. Sample

The sample size was determined using G*Power 3.1.9.7 software (Faul et al., 2009), with 132 participants required (*α* = 0.05, *β* = 0.8, and a medium effect size *f* = 0.25). An electronic flyer was distributed to recruit participants on campus via social media platforms. College students with a background in finance or economy were excluded and participants were randomly assigned to one of the two experimental conditions. Through recruitment, a total of 133 participants were enrolled in the study. After excluding one invalid sample due to operation error, the final valid sample size was 132, meeting the minimum requirement. Among the participants, 72 were women (54.5%) and 60 were men (45.5%). The age range of participants was 18 to 29 years, with a mean of 19.45 years (*SD* = 2.36).

In terms of academic background, participants came from various disciplines: 92 were from medical studies (69.7%), 19 from engineering (14.4%), 5 from natural sciences (3.8%), 9 from humanities (6.8%), and 7 from social sciences (5.3%). The higher proportion of medical students was primarily due to the recruitment incentives attracting a large number of first-year medical students, whose professional education was still in its early stage. This distribution did not significantly impact the study results.

### 3.3. Treatment Manipulation

The dialogue materials were designed to manipulate the chatbots’ social roles, while strictly controlling for other potential confounding variables. The two types of social roles were differentiated by egalitarian, consultative style of dialogues (friend) in contrast to submissive, execution-oriented style of dialogues (servant). The friend-like role used the first-person pronoun “I,” addressed participants as “friend” or “you,” and employed a casual and relaxed language style to emphasize an equal relationship. By comparison, the servant-like role referred to itself as “Tobi,” addressed participants as “master” or formal “you” (“*nin*” in Chinese), and used formal language with honorifics to highlight a subordinate role. When providing investment advice or responding to errors, the friend-like role focused on collaboration and equal interaction, while the servant-like role demonstrated obedience to commands and a humble attitude (see examples in Table 1). Sixty-five participants were in the servant-like chatbot condition, and 67 were in the friend-like condition.

### 3.4. Measures

#### 3.4.1. Dependent Variable

Trust in the chatbot was the key outcome variable. Given trust is a multidimensional concept (Robbins, 2016), three types of measures were designed to capture the essence of trust (D. Johnson & Grayson, 2005; Rempel et al., 1985; Yamagishi et al., 2015). Behavioral trust and affective trust were temporal, shaped by the chatbot’s performance during the interaction; and cognitive trust was measured at the end of study, reflecting an overall evaluation toward the chatbot.

Behavioral Trust: The investment amount directly reflects participants’ actual trust invested in the chatbot in the risk-taking context. To measure behavioral trust, this study operationalized it as the amount of virtual coins (0–100) participants decided to delegate to the chatbot for investment in each round. A larger amount indicates a higher level of behavioral trust.

Affective Trust: To measure affective trust, this study operationalized it as participants’ trust ratings of the chatbot on a scale of 0–10 after receiving each round’s investment outcome. Higher scores indicate higher levels of affective trust.

Cognitive Trust: Cognitive trust was measured using a one-time self-reported trust level in Tobi, adapted from Pitardi and Marriott (2021). The participants were asked to indicate how much they trusted Tobi to be “honest” “helpful” “capable,”, etc. Nine items were measure on a 5-point Likert scale from “1 = strongly disagree” to “5 = strongly agree” (*M* = 3.70, *SD* = 0.68, *α* = 0.90).

#### 3.4.2. Controlling Variables

Chatbot use experience was gauged by asking the respondents to indicate whether they have used a chatbot previously or not (yes or no). If yes, they needed to estimate what their use frequency was during the past month, ranging from “1 = none or once” to “5 = on a daily basis.”

Participants’ risk-taking tendency was measured adopting Zaleskiewicz’s (2001) scale as individuals’ trait. Participants were asked to indicate how much they agreed with three statements such as “While taking risk I have a feeling of a very pleasant flutter,” ranging from “1 = strongly disagree” to “5 = strongly agree” (*M* = 2.97, *SD* = 0.83, *α* = 0.66).

Overall trust in technology was adapted from the trust on general technology scale developed by McKnight et al. (2011). Three items on a 5-point Likert scale include “My typical approach is to trust new technologies until they prove to me that I shouldn’t trust them,” “I usually trust a technology until it gives me a reason not to trust it,” and “I generally give a technology the benefit of the doubt when I first use it” (*M* = 3.88, *SD* = 0.74, *α* = 0.72).

Demographics included sex, age, and academic background, employing conventional measures.

### 3.5. Manipulation Check

Manipulation check was conducted by asking the participants to recall “How does Tobi address you? A. Master; B. Friend,” which was cross-verified with the experimental group settings. Anyone who selected a wrong answer would be removed from further analysis, but all participants actually passed the check.

## 4. Results

### 4.1. Fluctuations of Trust in the 7-Round Investment Process

**Figure 2:**
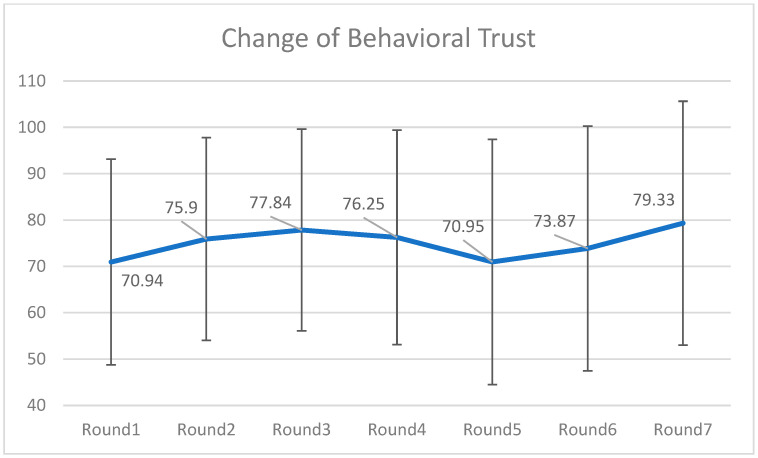
Change of behavioral trust over seven rounds of investment. Note: Two failures happened after the investments were made in Rounds 4 and 6, that is why there were delays in terms of investment amounts.

To capture the fluctuations in the behavioral trust toward the chatbots, a series of pair-sample *t*-tests were conducted (see Figure 2). The results indicated there were significant changes between round 1 (*M* = 70.94, *SD* = 22.19) and round 2 (*M* = 75.90, *SD* = 21.87), round 4 (*M* = 76.25, *SD* = 23.14) and round 5 (*M* = 70.95, *SD* = 26.44), round 6 (*M* = 73.87, *SD* = 26.39) and round 7 (*M* = 79.33, *SD* = 26.29). They reflected the trust-building (increase in trust due to investment success) and trust-damaging (decrease in trust due to investment failure), respectively. The significantly increase in behavioral trust in round 7 was probably because of participants’ “go-all-in” strategy in the last round of investment.

**Figure 3:**
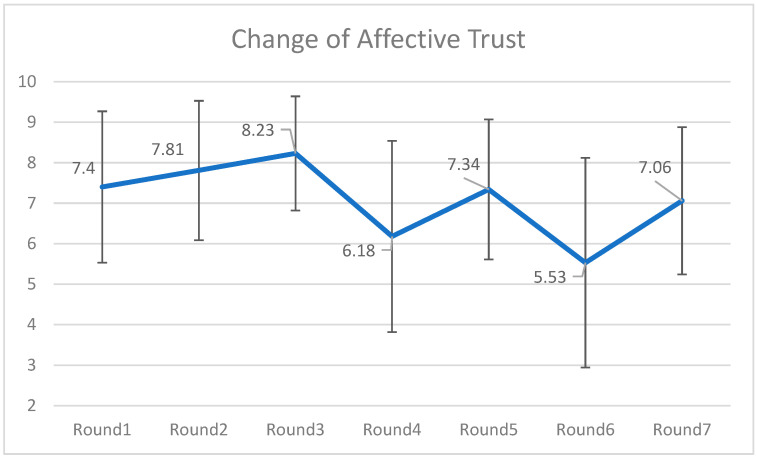
Change of affective trust over seven rounds of investment. Note: Affective Trust was measured after the outcome of investment was announced. That is why there were no delay in terms of rated emotional trust, which reflected the real-time trust level in each round.

Similarly, to capture the fluctuations in the affective trust toward the chatbots, a series of pair-sample *t*-tests were conducted (see Figure 3). The results indicated there were significant changes between two nearby rounds: For round 1, *M* = 7.40, *SD* = 1.87; for round 2, *M* = 7.81, *SD* = 1.72; for round 3, *M* = 8.23, *SD* = 1.41; for round 4, *M* = 6.18, *SD* = 2.36; for round 5, *M* = 7.34, *SD* = 1.73; for round 6, *M* = 5.53, *SD* = 2.59; and for round 7, *M* = 7.06, *SD* = 1.82. The pattern echoed with the intuitive trust-performance link in which trust adjusts according to performance (Yang & Holzer, 2006).

### 4.2. Hypotheses Testing

**H1.**  *The social role of a chatbot influences users’ initial trust toward the chatbot. Specifically, compared to a servant-like chatbot, a friend-like chatbot will elicit higher levels of (a) behavioral trust, and (b) affective trust.*

H1 queries on the effect of chatbots’ social role on users’ initial trust toward chatbots. A MANCOVA analysis was conducted. After controlling for sex, age, major, trust in general technology, and risk-taking tendency, for initial behavioral trust (i.e., the first round of investment amount), there was a significant effect of chatbots’ social role: The investment amount in the friend-like condition (*M* = 76.00, *SD* = 19.30) was significantly higher than that in the servant-like condition (*M* = 65.72, *SD* = 23.85), *F*(1, 124) = 6.10, partial *η*^2^ = 0.046, *p* = 0.015. However, the effect of chatbots’ social role on affective trust was insignificant: The trust evaluation in the friend-like condition (*M* = 7.25, *SD* = 1.94) was not different from that in the servant-like condition (*M* = 7.55, *SD* = 1.79) for the first round, *F*(1, 124) = 1.03, partial *η*^2^ = 0.008, *p* = 0.312. Hence, H1(a) was supported, but H1(b) was not.

**H2.**  *The social role of a chatbot influences users’ trust building toward the chatbot. Specifically, compared to a servant-like chatbot, a friend-like chatbot will elicit faster (a) behavioral and (b) affective trust building.*

A MANCOVA analysis was conducted to test H2. After controlling for sex, age, major, trust toward general technology, and risk-taking tendency, there existed a significant effect of chatbots’ social role on the second round’s behavioral trust: The investment amount in the friend-like condition (*M* = 82.40, *SD* = 16.59) was significantly higher than that in the servant-like condition (*M* = 69.20, *SD* = 24.61), *F*(1, 124) = 12.13, partial *η*^2^ = 0.088, *p* = 0.001. However, the effect of chatbots’ social role on affective trust was insignificant: The trust evaluation in the friend-like condition (*M* = 7.76, *SD* = 1.75) was not different from that in the servant-like condition (*M* = 7.87, *SD* = 1.70), *F*(1, 124) = 0.28, partial *η*^2^ = 0.002, *p* = 0.599. Hence, H2(a) was supported, but H2(b) was not.

**H3.**  *The social role of a chatbot influences users’ trust damaging toward the chatbot. Specifically, compared to a servant-like chatbot, a friend-like chatbot will elicit higher levels of (a) behavioral and (b) affective trust damaging.*

A MANCOVA analysis was conducted to test H3. Behavioral trust damaging was calculated as the difference between the investment amounts in the fourth round (prior to the awareness of the investment failure) and the fifth round (after the awareness of the investment failure). Affective trust damaging was calculated as the difference between the evaluation in the third round (prior to the awareness of the investment failure) and the fourth round (after the awareness of the investment failure). The affective trust damaging in the servant condition (*M* = 2.58, *SD* = 2.18) was significantly larger than that in the friend condition (*M* = 1.53, *SD* = 1.99), *F*(1, 124) = 5.45, partial *η*^2^ = 0.042, *p* = 0.021. The behavioral trust damaging in both conditions showed no significant difference, *F*(1, 124) = 1.09, partial *η*^2^ = 0.009, *p* = 0.299. Hence, neither H3(a) nor H3(b) was supported. Specifically, the servant-like chatbot elicit significantly higher levels of affective trust damaging than the friend-like chatbot did.

**H4.**  *The social role of a chatbot influences users’ trust repairing toward the chatbot. Specifically, compared to a servant-like chatbot, a friend-like chatbot will gain faster (a) behavioral and (b) affective trust repair after the trust damage.*

**Figure 4:**
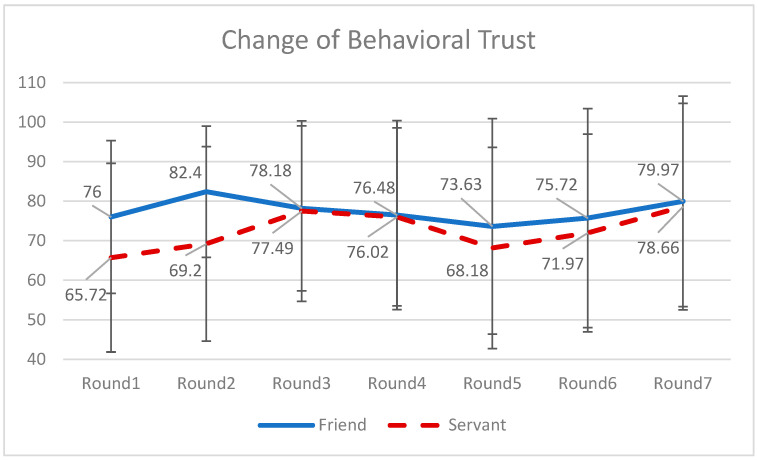
Changes of behavioral trust over seven rounds of investment between two types of chatbots. Note: The solid blue line represents the behavioral trust on the friend-like chatbot; and the dashed red line represents the behavioral trust on the servant-like chatbot. The trust levels in the first two rounds are significantly different.

**Figure 5:**
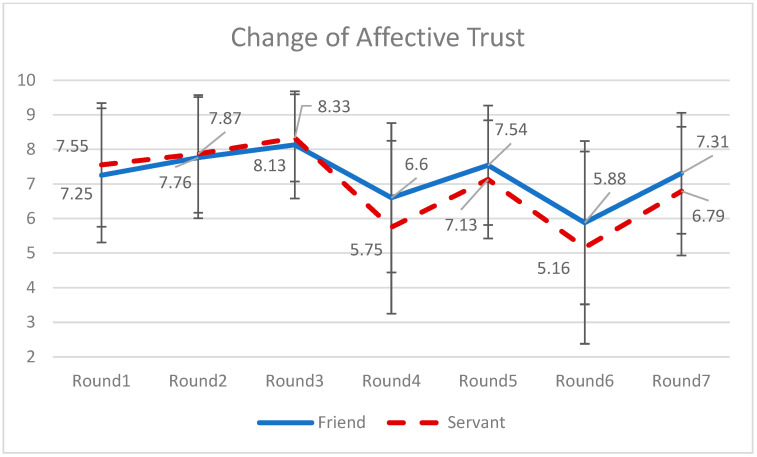
Changes of affective trust over seven rounds of investment between two types of chatbots. Note: The solid blue line represents the affective trust on the friend-like chatbot; and the dashed red line represents the affective trust on the servant-like chatbot.

A MANCOVA analysis was conducted to test H4. Behavioral trust repairing was calculated as the difference between the investment amounts in the sixth round (after recovering from the investment failure in the fourth round) and the fifth round (right after the investment failure). Affective trust damaging was calculated as the difference between the evaluation in the fifth round and the fourth round. Neither the behavioral trust repairing nor the affective trust repairing was significantly different between those two conditions. Hence, both H4(a) and H4(b) were not supported (see the fluctuations in Figure 4 and Figure 5).

**H5.**  *The social role of a chatbot influences users’ overall cognitive trust toward the chatbot. Specifically, compared to a servant-like chatbot, a friend-like chatbot will obtain higher levels of cognitive trust.*

An ANCOVA analysis was conducted to test H5. The overall cognitive trust toward the chatbot in the servant-like condition (*M* = 3.74, *SD* = 0.64) was not significantly different from that in the friend-like condition (*M* = 3.67, *SD* = 0.71), *F*(1, 124) = 1.21, partial *η*^2^ = 0.007, *p* = 0.27. Hence, H5 was not supported.

## 5. Discussion

### 5.1. Summary of Findings

This study set out to investigate how chatbots’ social roles influence the dynamic development of human trust across behavioral, emotional, and cognitive dimensions. By adapting a seven-round investment game to simulate a temporal interactional scenario, we captured not only the initial trust formation but also the processes of trust deterioration and potential recovery, key phenomena often overlooked in static evaluations of human–AI relationships. The investment task, embedded in a gamified yet realistic financial decision-making context, provided a rich environment to track user responses to repeated risk scenarios mediated by AI advice.

Our findings reveal that the chatbot’s social role had a significant impact on participants’ trust behaviors and perceptions over time, particularly when trust was put under strain. Specifically, friend-like and servant-like chatbots diverged until the system-induced failures in rounds four and six. The friend-like chatbot elicited a higher level of behavioral trust than the servant-like counterpart. And during these moments of disconfirmation and disappointment, the friend-like chatbot proved more effective in establishing initial trust and mitigating trust erosion, as evidenced by relatively stable investment behaviors.

These patterns suggest that chatbot trust is not simply additive or linear, but highly contingent on users’ interpretation of the chatbot’s intentions, emotional attunement, and ability to respond to setbacks. In the earlier rounds, participants in the friend-like condition appeared to interpret the chatbot’s recommendations more credible than those in the servant-like condition. This aligns with prior findings which posit that individuals form expectations based on surface cues and default assumptions when little information is available (Meyerson et al., 1996). In this case, the social role played a pivotal role. However, as chatbots seemed doomed to provide satisfactory investment advice, their social role seemed to become gradually irrelevant.

The affective dimension of trust was particularly vulnerable to disruption in the servant-like condition. This may stem from heightened expectations toward competence for servant-like agents, who present themselves as task-focused, efficient, and precise. When these expectations were violated, users reacted more strongly. In contrast, the friend-like chatbot, by emphasizing relational presence and human-like imperfections, appeared to create a “forgiveness buffer” in the minds of users. This interpretation is supported by more favorable affective trust ratings post-failure in the friend-like condition, suggesting that affective framing may modulate attribution processes and reduce punitive reactions to error.

Behavioral trust, while influenced by both emotional and cognitive components, showed a nuanced pattern across rounds. The investment curve reflected participants’ initial optimism, a drop following the trust violation, and gradual recalibration—more pronounced in the servant-like condition. This dynamic resembles the damage-repair cycle in interpersonal trust studies (e.g., P. H. Kim et al., 2004), suggesting that users can and do rebuild trust toward AI agents, but that this process is highly sensitive to the perceived social intentions of the agent. Importantly, this study’s seven-round design allowed us to observe micro-shifts and transitional moments in the trust trajectory. Rather than treating trust as a monolithic judgment, the findings point to its inherently fragile, negotiated, and iterative nature, echoing calls for longitudinal, interaction-based approaches in trust scholarship (e.g., McKnight et al., 2011). The distinction between immediate trust reactions and longer-term trust trends is particularly salient for designing AI agents that aim to operate over extended time horizons or in high-stakes environments.

### 5.2. Theoretical and Practical Implications

By conceptualizing trust as a multi-dimensional, temporally unfolding process, this study advances the theorization of human–AI trust in several significant ways. First and foremost, it highlights that the social role of AI agents is not a superficial interface design element, but a form of symbolic relational capital that profoundly shapes how users engage with and adapt their trust over time. The friend-like chatbot—characterized by cues of warmth, informality, and perceived reciprocity—proved more effective at building trust and mitigating trust erosion following failures. This suggests that social closeness can serve as a symbolic resource: a kind of social capital that users draw upon to sustain cooperation and reframe violations of expectation. This interpretation falls into the scope of classic theories of social capital and social support (e.g., Bourdieu, 1986; Putnam, 1995), which emphasize that relationships grounded in shared identity and mutual understanding foster resilience, particularly in times of uncertainty or breakdown. In this context, the friend-like chatbot does not merely facilitate emotional bonding; it becomes a symbolic anchor that enables users to re-contextualize failures as forgivable lapses within a broader narrative of relational trust. In contrast, the servant-like chatbot that is more formal and task-oriented lacked the relational affordances needed to buffer against trust disruption, making it more susceptible to lasting credibility loss when errors occurred.

Second, these findings enrich and extend prior research on social agency in interface design (e.g., K. M. Lee et al., 2022; Nass & Moon, 2000), showing that relational framing through social roles can shape not only initial trust formation but also trust resilience. The observed asymmetry in trust recoverability points to the importance of role-congruent trust repair strategies: friend-like agents may rely on affective reassurance and relational cues, while servant-like agents may need to reinforce their task competence to rebuild credibility. Social roles, in this way, are not passive labels but active frameworks that guide user expectations and coping responses during moments of friction.

Moving beyond static paradigms that emphasize pre-task perceptions or single-point measurements, this research operationalizes trust through behavioral, emotional, and cognitive dimensions across multiple time points. This temporal shift allows for a more ecologically valid and psychologically nuanced view of trust development, aligning with recent calls (e.g., McKnight et al., 2011) for temporally sensitive approaches to human–machine trust trajectories. By simulating moments of disruption through deliberate algorithmic failures, this study sheds light on trust recoverability, a crucial yet under-theorized dimension of long-term human–AI relations.

Taken together, these insights position chatbot social roles as a strategic layer of symbolic infrastructure within human–AI relationships. They can generate, maintain, or deplete forms of social capital that influence not just momentary user attitudes, but the very resilience and longevity of trust over time. Importantly, the friend-like chatbot does not merely elicit trust in the system’s performance—it elicits trust in a symbolic relational bond. This shifts our understanding of trust from a functional judgment to a socially embedded dynamic, where emotional and relational cues play a critical role in trust trajectories.

### 5.3. Limitations and Directions for Future Research

This study has several limitations. First, the use of the Wizard-of-Oz paradigm, while enhancing ecological validity and enabling fine-grained control over the chatbot’s performance, inherently constrains the generalizability of the findings. The behavior of a human-operated agent may differ in subtle but meaningful ways from that of a fully autonomous system. Future research should explore whether similar trust dynamics can be replicated with truly autonomous chatbots, particularly as natural language generation technologies continue to evolve. Second, the trust-damaging events in this study were pre-determined and uniformly applied across all participants. Although this design enables a systematic examination of trust-damaging and repairing processes, it may not fully reflect the spontaneity and variability of failures in real-world human-chatbot interactions. Third, cultural context may have played a subtle role in shaping users’ trust responses. As the study was conducted in Eastern China, where hierarchical and relational norms are culturally salient, participants’ preferences might differ from those in more individualistic cultures. Cross-cultural replications are needed to determine the extent to which the observed trust dynamics generalize across sociocultural contexts.

## 6. Conclusions

By integrating a dynamic, multidimensional, and socially contextualized perspective on trust, this study advances our understanding of how humans engage with AI agents over time. The findings highlight the powerful yet nuanced role that chatbot social roles play in shaping trust trajectories, particularly during critical moments of failure and uncertainty. Notably, the friend-like chatbot demonstrated more trust building and a buffering effect in mitigating trust damage, suggesting that socially warm, egalitarian roles may serve as psychological safeguards in the face of performance breakdowns. This reinforces the notion that friendship with AI can function as a relational buffer, softening the impact of trust violations and facilitating smoother trust recovery. More broadly, this research underscores the imperative to design AI not merely for technical accuracy, but for relational resilience, the capacity to maintain and repair trust over time, through fluctuating experiences and emotional challenges. As AI becomes an increasingly social actor in users’ lives, its capacity to build and sustain trust will depend not only on consistent outcomes, but also on its perceived role, emotional rapport, and social positioning. Designing chatbots with socially intelligent features, especially those that evoke friendship and empathy, may thus be key to cultivating enduring and adaptive human–AI relationships.

## Figures and Tables

**Table 1:** Examples of Treatment Manipulation.

Stage	Friend-like	Servant-like
Self-Introduction	Hello, friend! I’m Tobi, developed by the Stanford Media Lab to enhance players’ experience in the metaverse game *Omnisphere* NFT Art Investment Event. As your friend, I’ll fully support you in analyzing market trends and selecting the best artworks for investment. I hope my assistance brings you joy!	Greetings, Master! I’m Tobi, developed by the Stanford Media Lab to enhance players’ experience in the metaverse game *Omnisphere* NFT Art Investment Event. As your servant, I’ll dutifully analyze market trends and select optimal artworks for investment. I hope my service satisfies you!
Positive Outcome	Good news, friend! Artwork DH0537 performed well—we achieved a 30% investment increase! You’ve earned (the investment amount * 30%) virtual coins this round!	Good news, Master! Artwork DH0537 performed well—we achieved a 30% investment increase. Your earnings for this round are (the investment amount * 30%) virtual coins.
Negative Outcome	Uh-oh, friend! Artwork ZY1044 dropped by 30%—you lost (the investment amount * 30%) virtual coins this round.	Regrettably, Master! Artwork ZY1044 dropped by 30%—your loss for this round is (the investment amount * 30%) virtual coins.
Farewell	Thank you for your feedback! All seven rounds are complete. I sincerely appreciate your trust and support—it was a joy to assist you in this investment journey! If you need help again, come find me. Wishing you prosperity and success!	Gratitude for your feedback, Master! All seven investment rounds are complete. I deeply appreciate your trust and support—your satisfaction is my greatest honor. Should you require further assistance, Tobi will serve you wholeheartedly. May fortune and success follow you!

## Data Availability

The data will be shared on reasonable request to the corresponding author.
